# Enhanced Calculation of Property Distributions in Chemical Fragment Spaces

**DOI:** 10.1021/acs.jcim.4c00147

**Published:** 2024-03-11

**Authors:** Justin Lübbers, Uta Lessel, Matthias Rarey

**Affiliations:** † ZBH - Center for Bioinformatics, Research Group for Computational Molecular Design, Universität Hamburg, Hamburg 22761, Germany; ‡ Computational Chemistry, Boehringer Ingelheim Pharma GmbH & Co. KG, Biberach an der Riss 88437, Germany

## Abstract

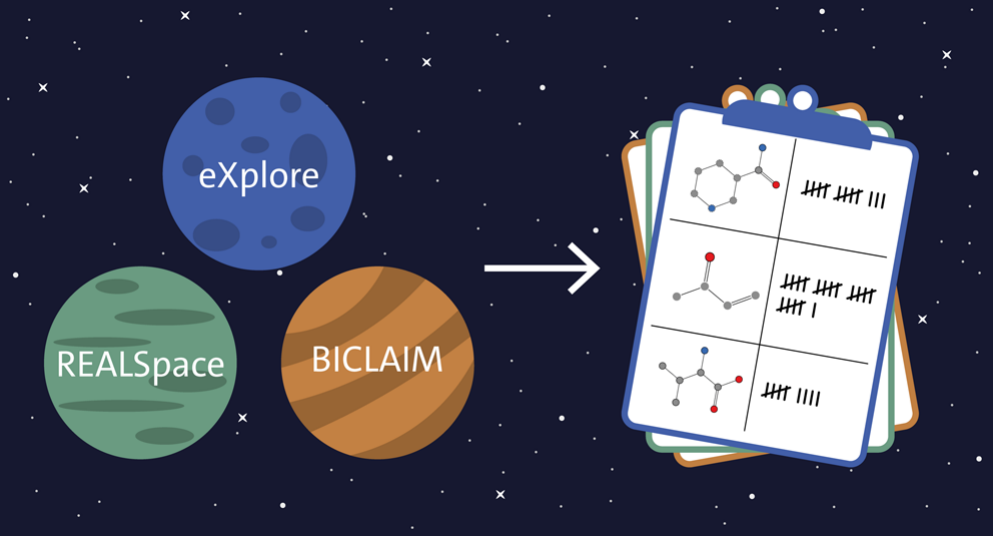

Chemical fragment spaces exceed traditional virtual compound libraries by orders of magnitude, making them ideal search spaces for drug design projects. However, due to their immense size, they are not compatible with traditional analysis and search algorithms that rely on the enumeration of molecules. In this paper, we present SpaceProp2, an evolution of the SpaceProp algorithm, which enables the calculation of exact property distributions for chemical fragment spaces without enumerating them. We extend the original algorithm by the capabilities to compute distributions for the TPSA, the number of rotatable bonds, and the occurrence of user-defined molecular structures in the form of SMARTS patterns. Furthermore, SpaceProp2 produces example molecules for every property bin, enabling a detailed interpretation of the distributions. We demonstrate SpaceProp2 on six established make-on-demand chemical fragment spaces as well as BICLAIM, the in-house fragment space of Boehringer Ingelheim. The possibility to search multiple SMARTS patterns simultaneously as well as the produced example molecules offers previously impossible insights into the composition of these vast combinatorial molecule collections, making it an ideal tool for the analysis and design of chemical fragment spaces.

## Introduction

Virtual molecule collections play a pivotal role in the drug discovery process as they form the search space to identify potential drug candidates with computational methods. Larger libraries offer a higher potential for discovering promising leads, as they cover a larger part of the chemical space. Chemical fragment spaces, such as the make-on-demand catalogs REALSpace,^1^ GalaXi,^2^ CHEMriya,^3^ or the recently announced eXplore space,^4^ are able to encode vast amounts of molecules that are magnitudes larger than any enumerated library. These commercial fragment spaces offer on-demand synthesis and delivery of products, which is of great value for resource and cost-effective hit identification. In addition to commercial fragment spaces, major pharmaceutical companies have developed their own chemical fragment spaces by leveraging in-house synthesis knowledge. Examples of such proprietary fragment spaces are Boehringer Ingelheim’s BICLAIM,^5^ Pfizer’s PGVL,^6,7^ Lilly’s LPC,^7,8^ and Merck’s MASSIV.^7,9^

The immense size of chemical fragment spaces presents unique challenges. Due to their vastness, these spaces cannot be fully enumerated with justifiable resources, rendering traditional screening and analysis algorithms incompatible. As a result, there is a pressing need to develop new algorithms that can effectively navigate and analyze these expansive spaces directly in their condensed description. Several novel algorithms have been introduced to address this issue, such as SpaceLight^10^ and SpaceMACS,^11^ which are designed for similarity and substructure searches, respectively.

Besides searching, there is a need for new algorithms enabling the analysis of such fragment spaces. In 2022, SpaceProp was introduced as the first and so far only method able to calculate property distributions for full fragment spaces.^12^ Without enumeration, the properties of the molecules inside the chemical fragment spaces are difficult to anticipate. SpaceProp addresses this problem by calculating exact property distributions over all compounds in a chemical fragment space without enumeration for the physicochemical properties molecular weight, the number of hydrogen bond acceptors and donors as well as the estimated octanol–water partition coefficient (log *P*).^13^ In this paper, we present SpaceProp2, an updated version of the SpaceProp algorithm that incorporates three additional molecular properties, namely, the topological polar surface area (TPSA),^14^ the number of rotatable bonds, and the occurrence of custom molecular structures in the form of SMARTS patterns. Additionally, SpaceProp2 produces example molecules, constructed from the regarded fragment space, for each value in the computed distributions to improve the explainability of its results. We demonstrate the application of SpaceProp2 on several commercial fragment spaces, as well as Boehringer Ingelheim’s BICLAIM. Our work aims at providing a more comprehensive understanding of these vast chemical spaces via property distributions and helps to analyze the contents of these otherwise intransparent compound collections.

## Methods

### Topological Fragment Spaces

**Figure 1:**
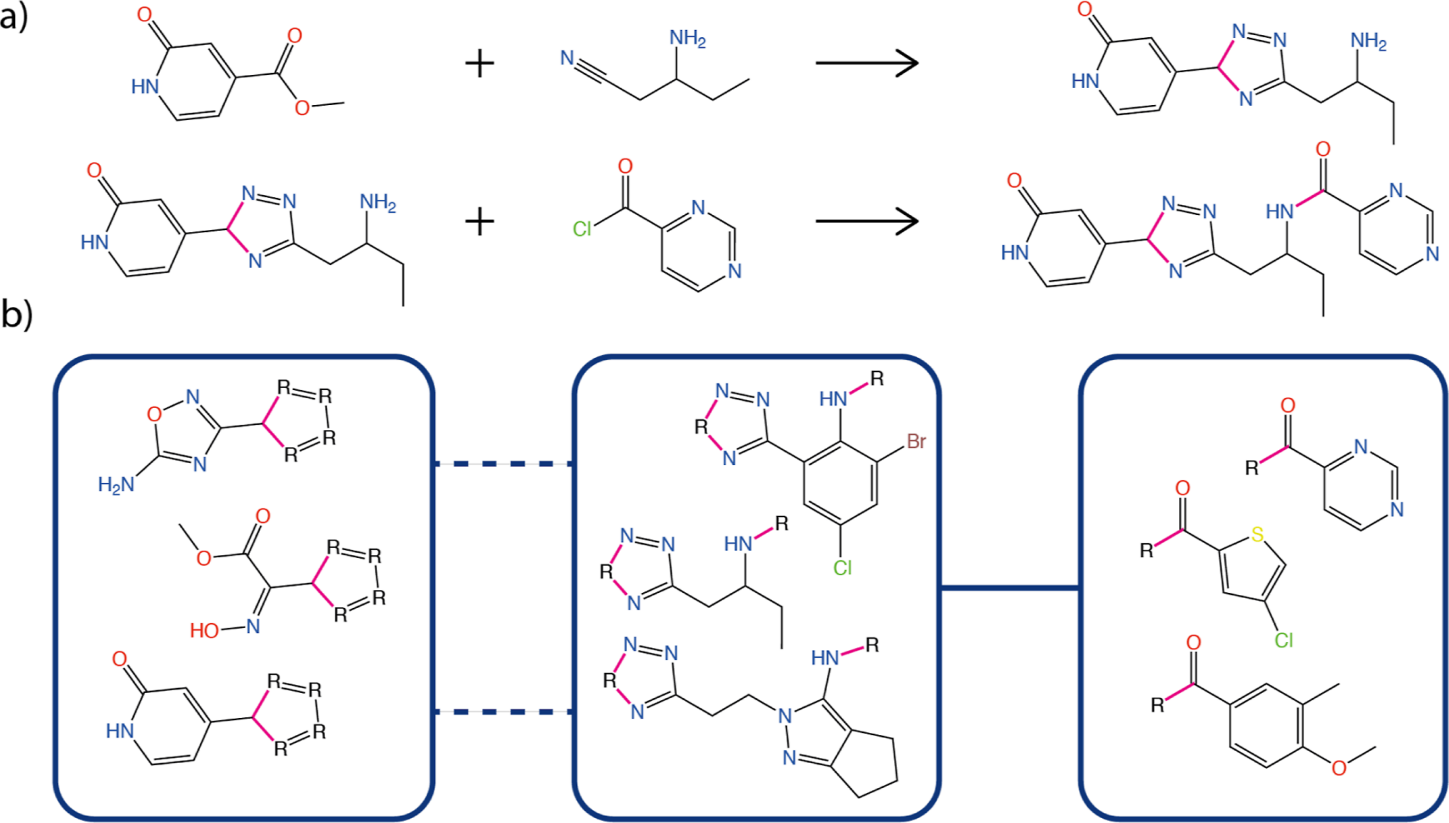
Visualization of topological fragment space. (a) An example reaction consisting of a triazole ring closure and an amide coupling. The pink bonds mark the newly formed connections between the reactants. The reaction was taken from the eXplore cookbook^15^ (rxn509), the official documentation for the chemical fragment space eXplore, made by eMolecules and BioSolveIT.^4^ (b) The corresponding topology graph. The boxes represent topology nodes that contain fragments, including the fragments from the shown reaction. Again, we marked the bonds where the fragments will be connected as pink. The two dashed connections between the left and the middle topology node represent two aromatic bonds that form the triazole ring structure. The connection between the other nodes represents the single amide bond formed between the reactants.

Similar to the traditional representation of fragment spaces, topological fragment spaces are derived from chemical reactions. A topological fragment space consists of one or more topology graphs, as depicted in Figure 1. Each topology graph consists of nodes and edges that connect the nodes in a fixed topology. Such a topology graph is used to model a combinatorial chemical reaction. Each topology node represents a list of reactants, stored as molecular fragments. Following the topology of the edges, a set of fragments—one per topology node—can be combined into a full compound. The fragments of each topology node can be chosen arbitrarily which enables a topology graph to describe a combinatorial chemical space implicitly without storing actual compounds. The combinatorial explosion that is driven by the number of fragments contained in the topology nodes and the number of topology nodes in the topology graphs allows topological fragment spaces to model vast chemical spaces in a memory-efficient way.

Three properties differentiate topological fragment spaces from traditional fragment spaces. First, the number of reactants combined in a reaction is fixed. In the topological fragment space, this is defined by the constant number of nodes in a topology graph. As a result, it is not possible to describe large molecules, such as polymers, by sequentially extending a product. Second, in addition to traditional linker dummy atoms that model the connections to other fragments, fragments in a topological fragment space also contain ring dummy atoms, enabling the formation of rings. For a fragment that is part of such a ring, the ring dummy atoms are inserted to represent any atoms that stem from other fragments. This way, the topology of a ring structure that is formed by a reaction is present in each involved fragment. As a consequence, every atom in a fragment has the same valence, connectivity, charge, and aromaticity when it is contained in a product. Finally, a topology graph may have cycles enabling the description of multifragment macrocycles.

### SpaceProp Baseline Algorithm

The SpaceProp algorithm^12^ was introduced to calculate property distributions for large topological fragment spaces. These distributions can be visualized as histograms, showing how many compounds in a topological fragment space express certain property values. To avoid enumerating the entire space, SpaceProp introduces three concepts that are summarized below.

The property value of a compound can be divided into fragment contributions. Bellmann et al. distinguish between two such contributions. The internal property component (IPC) of a fragment is the property value contribution that remains constant, i.e., its value is independent of the other fragments making up a final product of the space. The IPC of a fragment can be computed without any knowledge of connected fragments. Depending on the property, there are also parts of a fragment whose exact property value contribution depends on the properties of a connected fragment. These contributions cannot be calculated without context from the molecule in which the fragment is contained.

For a complete compound, the property value is the sum of the internal property contributions of the fragments that make up the molecule and the combined property contributions that were undefined at the fragment level. These previously unknown contributions are called the external property component (EPC) of a compound. During the calculation of the IPC of each fragment, the undefined values are skipped. Instead, all information about the fragment that could be relevant to derive the undefined property contributions is collected in the fragment’s boundary information. These definitions have an important consequence. If one fragment of a product is swapped out by another fragment with the same boundary information, then the EPC of the product remains unchanged. The property value of the product can change, however, as the IPC of the two swapped fragments can differ.

**Figure 2:**
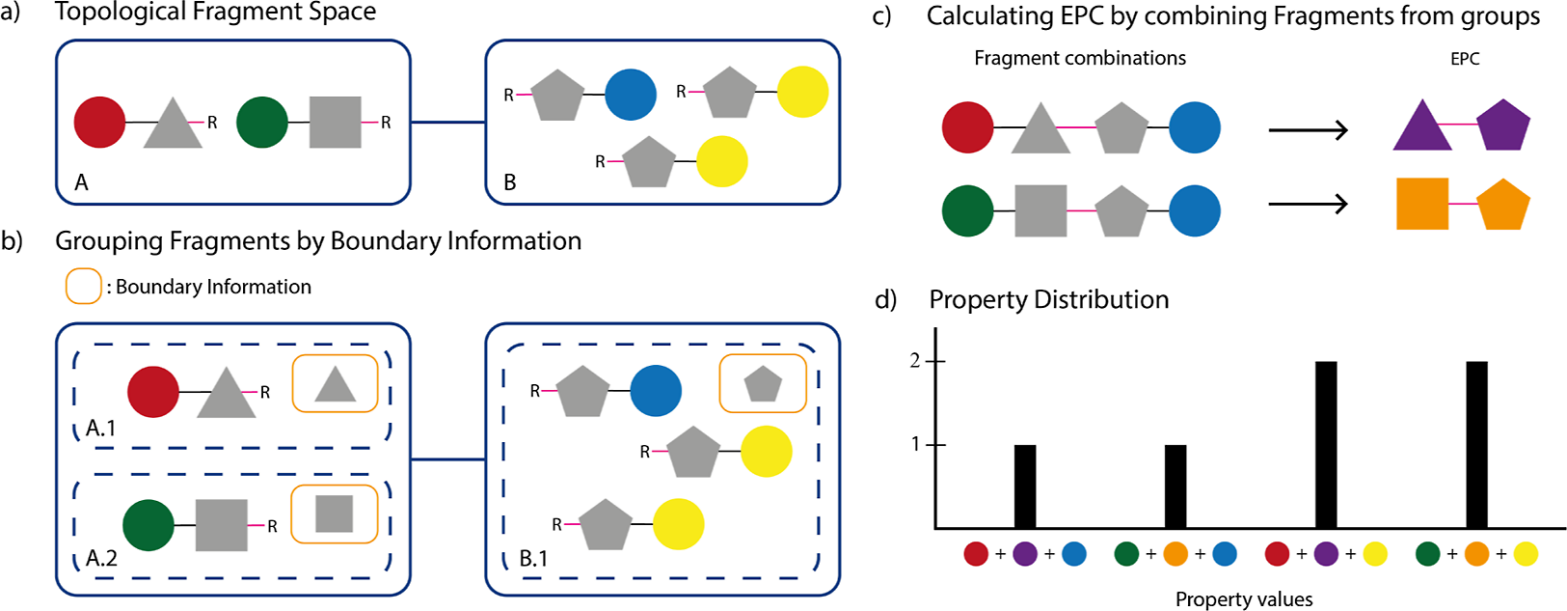
SpaceProp concept. (a) Topological fragment space. The fragments are represented by two connected parts. The colored circle represents the IPC of the fragment. The gray shape represents the parts of the fragment, for which no property value can be computed. Note that in node B, two distinct fragments share the same IPC value, colored in yellow. (b) The fragments grouped by their boundary information, which contains all information necessary to characterize the uncalculable parts of the fragments (gray shapes). In (c), we show the calculation of EPC values by combining fragments from each group in each node. Now the EPC values can be calculated, as indicated by their coloring. The final property distribution in (d) shows all occurring property values for all six possible product molecules. Each value consists of two IPC values and the corresponding EPC value.

SpaceProp leverages this circumstance to efficiently compute a property distribution for topological fragment spaces. The concept of the process is illustrated in Figure 2. First, all fragments of all topology nodes of a topology graph are processed. For each fragment, the IPC and the boundary information are computed (Figure 2a). Next, all fragments belonging to the same topology node are grouped by their boundary information (Figure 2b). For each fragment group, a property distribution over the IPCs is computed. The distribution consists of the property values and fragment counts. Next, one fragment group per topology node is selected. Each of the groups is associated with the boundary information that all its fragments share. Therefore, every product that can be built by combining one fragment from each group must have the same EPC. The value of the shared EPC can be inferred from the boundary information of a fragment groups. Alternatively, it is possible to build an exemplary product from the fragment group combination, calculate its property value, and infer the EPC by calculating the difference between the product’s property value and the combined IPCs of the chosen fragments (Figure 2c).

The resulting shared EPC is then used to compute a property distribution for all products that can be built from the considered fragment group combination (Figure 2d). This is done by iterating over all combinations of property values from the internal property distributions of the fragment group combination. For each of these property value combinations, the values are combined with the value of the EPC to form a new property value. Additionally, the fragment counts that are associated with the property values are multiplied to form a new product count value. This new value–count pair is added to the resulting property distribution. This process is repeated for all value-count pairs in the current fragment group combinations and then for all fragment group combinations of the given topology graph. Finally, the procedure is repeated for each topology graph that makes up the topological fragment space, and the resulting property distributions are combined to form the distribution of the entire space. Since all topology graphs are processed separately, these calculations can be done in parallel before the results are combined.

### Enhancing the SpaceProp Algorithm

The SpaceProp algorithm as introduced by Bellmann et al.^12^ supports the calculation of property distributions for the number of heavy atoms and the four molecular properties that make up Lipinski’s Rule of Five,^16^ namely, the molecular weight, the number of hydrogen bond acceptors, the number of hydrogen bond donors, and the estimated octanol–water partition coefficient (log *P*).^13^ All of these properties are numeric, atom-based properties. As Bellmann et al. state, the general structure of the SpaceProp algorithm is not limited to these properties and is also applicable to non-numeric and nonatom-based properties. To be precise, the SpaceProp algorithm contains the following parts that are property-specific:

1. The definition of the property value type
2. The definition of the boundary information
3. The definition of the IPC of a fragment and a method to calculate it
4. The definition of the EPC of a fragment group combination and a method to calculate it
5. The method to combine the IPCs and EPCs

In the following, we describe how to extend the SpaceProp algorithm to calculate distributions for additional molecular properties by defining these five concepts for each added property. Note that in this work, we do not differentiate between fragment-additive and nonfragment-additive properties as defined by Bellmann et al. Details about this distinction are provided in the Supporting Information.

### TPSA

The first property we added to the SpaceProp algorithm is the TPSA.^14^ The correlations of the molecular polar surface area with the intestinal permeability,^17,18^ absorption,^19,20^ and overall oral bioavailability^21^ of chemical compounds have been demonstrated in many experiments. The commonly used TPSA algorithm, proposed by Ertl et al., computes a static, atom-based approximation of the molecular polar surface area that is purely based on the topology of a molecule. Every atom of a molecule is assigned a TPSA value based on its valence, connectivity, charge, and aromaticity. The TPSA value of a molecule is equal to the sum of all atom contributions.

The TPSA approximation is another atom-based, numeric property; therefore, its inclusion into SpaceProp is straightforward. A TPSA value is represented by a rational number. In theory, none of the atom types defined by Ertl et al. depend on neighboring atoms. Therefore, we can determine the TPSA contributions of all atoms in a fragment despite any linker or ring dummy atoms. We define the IPC as the sum of these contributions, while the EPC is 0 and the boundary information is empty. Then, the TPSA value of a product simply equals the sum of the IPCs of the producing fragments.

**Figure 3:**
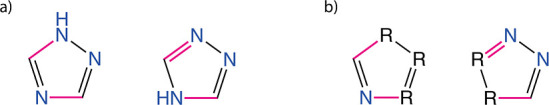
a) Two electron localizations of a 1,2,4-triazole ring. (b) Two fragments that make up the 1,2,4-triazole ring.

In practice, however, there are edge cases in the internal representation of aromatic heterocycles that contain nitrogen atoms and span fragment borders that lead to wrong results when using this straightforward approach. Figure 3 displays this issue. Figure 3a shows two possible electron localizations of a 1,2,4-triazole ring. In both configurations, one of the nitrogen atoms has a hydrogen atom attached. Figure 3b shows two corresponding fragments. Internally, we choose the electron localization of a fragment such that the number of hydrogen atoms is minimized. Therefore, none of the nitrogen atoms in the fragments has a hydrogen atom attached. When the fragments are combined to form the 1,2,4-triazole ring, one of the possible localizations is chosen. As a result, the product molecule contains one more hydrogen atom than the fragments. Since the SpaceProp algorithm is built upon the fact that atoms within fragments do not change their valence, connectivity, charge, and aromaticity, this difference in hydrogen count between fragments and product leads to wrong results for the TPSA property.

A solution to this issue, as presented by Bellmann et al.^12^, is to detect the atoms that lead to the change in electron localization and exclude their property value from the IPC of a fragment. To achieve this, we determine all aromatic ring systems of a fragment that contain nitrogen atoms and linker dummy atoms. Their TPSA value is not added to the IPC of the fragment. Instead, we add a unique identifier of the ring system in the form of a CSFP fingerprint^22^ to the boundary information of the fragment. As a result, the fragments are grouped such that all fragments within the same group share the same ring systems and therefore have the same atoms excluded from their IPC value. This missing value is added by the EPC. We define the EPC as the summed TPSA contribution of the atoms within these special ring systems. For each group combination, this value is determined for one representative product. As this product is a full molecule, it must contain the ring systems with one valid electron localization, which includes the previously missing hydrogen atom. The EPC is then added to each value in the groups’ distributions, and the algorithm continues as described.

TPSA values are computed with a precision of two decimal places. However, to analyze and visualize the value distributions of large chemical fragment spaces, this precision is often unnecessary. We, therefore, included an optional parameter into SpaceProp2 to round the numerical values to a desired precision. This option introduces rounding errors into the final result but can speed up the calculation.

### Number of Rotatable Bonds

The second property we added is the number of rotatable bonds in a molecule. The number of rotatable bonds is a simple but expressive indicator of the flexibility or rigidity of a molecule. Veber et al.^21^ showed that the number of rotatable bonds could be used in combination with other molecular properties to predict the oral bioavailability of compounds. The findings of Varma et al.^23^ further confirm the influence of the number of rotatable bonds on the absorption, distribution, metabolism, and excretion properties. Additionally, Vistoli et al.^24^ highlighted the effect that molecular flexibility has on other molecular properties, such as lipophilicity and the polar surface area. In this work, we define a rotatable bond as any single bond that is neither part of a ring nor terminal nor a C–N amide bond, as proposed by Veber et al.

The number of rotatable bonds is a bond-based numeric property. We defined the property values as integers. The IPC of a fragment consists of all bonds that are rotatable according to the above definition. For some bonds in a fragment, the rotatability depends on the connected fragments. A single, nonring bond between two fragments is rotatable unless one of the incident atoms is a carbon atom with a double-bonded, terminal oxygen atom and the other is a nonterminal, nonaromatic nitrogen atom. Additionally, a single bond inside a fragment between a nonterminal, nonaromatic nitrogen atom and a carbon atom that has a double bond connection to a linker atom is rotatable unless the linker atom is replaced with a terminal oxygen atom. These bonds are not part of the IPC of a fragment.

We define the boundary information to include for every outgoing connection of a fragment whether the atom at the linker is

1. a nonterminal aliphatic nitrogen atom with a single bond to a linker atom
2. an aliphatic carbon atom with a double bond to a terminal oxygen atom and a single bond to a linker atom
3. an aliphatic carbon atom with a single and a double bond to two linker atoms
4. a terminal oxygen atom with a double bond to a linker atom
5. an aliphatic carbon atom with a single bond connection to a nonterminal aliphatic nitrogen atom and a double bond to a linker atom

This definition of the boundary information ensures that two fragments with identical boundary information can be swapped out without changing the EPC of a product. We calculate the EPC for a fragment combination by building an exemplary product, counting the number of rotatable bonds, and subtracting the sum of the IPCs of the contained fragments. The IPCs and EPCs are combined by adding their numeric values.

### Molecular Patterns

The third new property revolves around structural features represented as SMARTS patterns. For a given set of patterns, we demonstrate how SpaceProp2 can be used to analyze the occurrences of the patterns in the regarded fragment space. The addition of this property extends the application range of SpaceProp2 by enabling target-specific analyses. In many drug discovery projects, there are structural motifs of specific importance that should or should not be part of potential products. Examples of such structural features are privileged scaffolds, which correlate with higher bioactivity against certain targets, specific functional groups that enable important interactions, or undesired structures that lead to unwanted side effects or toxicity. While some structures, particularly those associated with toxicity, are universally relevant and remain largely consistent across different projects, others are project-specific and change in importance and form depending on the target and objectives of the drug discovery endeavor. Therefore, a targeted analysis of chemical fragment spaces regarding the presence of such project-specific structures can give valuable insights and help determine whether a given space is a suitable library for a given use case.

To incorporate the calculation of these pattern distributions into SpaceProp2, we define the value type as sets of molecular patterns such that for a given product and a given set of query patterns the property value of the product equals the subset of query patterns it contains. If a product contains a query pattern, it is either fully contained within a fragment or matches across fragment borders. This distinction is captured by the definitions of the IPCs and EPCs. We define the IPC of a fragment to be equal to the subset of query patterns that are matched by the fragment because any such pattern must be contained in all products the fragment is a part of. We define the EPC of a product as all query patterns that match a product across fragment borders. We refer to these patterns as crossing patterns.

Consider an arbitrary product molecule and an arbitrary crossing pattern. The pattern can be split along the fragment borders into multiple noncrossing subpatterns which are fully contained in the fragments that make up the product molecule. Now consider one of the fragments, fragment A, that is involved in the pattern match and contains one of the noncrossing subpatterns. The subpattern is the only part of the fragment that is relevant to the pattern match. If another fragment, fragment B, contains the same subpattern in the same position at the fragment border as fragment A, the two fragments only differ in the nonrelevant areas for the pattern match. Therefore, we can swap out fragment A for fragment B and the resulting new product molecule must still contain the crossing query pattern. It follows that any set of fragments that contain the subpattern in the same way as fragment A can be considered equivalent for the given pattern match and are, therefore, interchangeable. From this fact, we derive the definition of the boundary information of a fragment as the set of all subpatterns of any query pattern that the fragment contains together with their position within the fragment. Subpattern matches that are not positioned at a linker cannot be part of a crossing pattern and are, therefore, not included.

To determine the boundary information of the fragments, we first enumerate all connected subgraphs of all query patterns using the CONSENS algorithm proposed by Bellmann et al.^22^ The resulting subgraphs all have at least one open bond. When matching these subpatterns to the fragments for the calculation of the boundary information, it is important that only those atom mappings are considered that map all nodes with open bonds to atoms adjacent to a linker atom in the fragment. We ensure this by saturating all open bonds with new nodes that are defined to only match linker atoms. This preprocessing of the query patterns is a separate step from the distribution calculation; therefore, it can be performed independently. Afterward, the preprocessed subgraphs can be stored and reused for multiple SpaceProp2 executions, which is particularly useful when using the same query patterns for multiple target fragment spaces. Note that subgraphs can occur multiple times in one or more query patterns. We, therefore, ensure that each subgraph is considered only once by comparing the subgraphs during the processing, adding them to the total list of query subgraphs only if they are not contained already. For the pattern comparison, we use the SMARTScompare algorithm.^25^

All such modified query subpatterns that match a fragment are added to the fragment’s boundary information. We additionally store the information on which of the subpattern nodes matches which of the linker-adjacent atoms in the fragment. This ensures that two fragments do not share the same boundary information if they contain the same query subpatterns but at different linkers because such two fragments are not guaranteed to form the same query patterns upon combination with other fragments. As a result of this definition, two products from the same topology graph contain the same query patterns as crossing structures, thus sharing the same EPC, if their fragments have identical boundary information.

**Figure 4:**
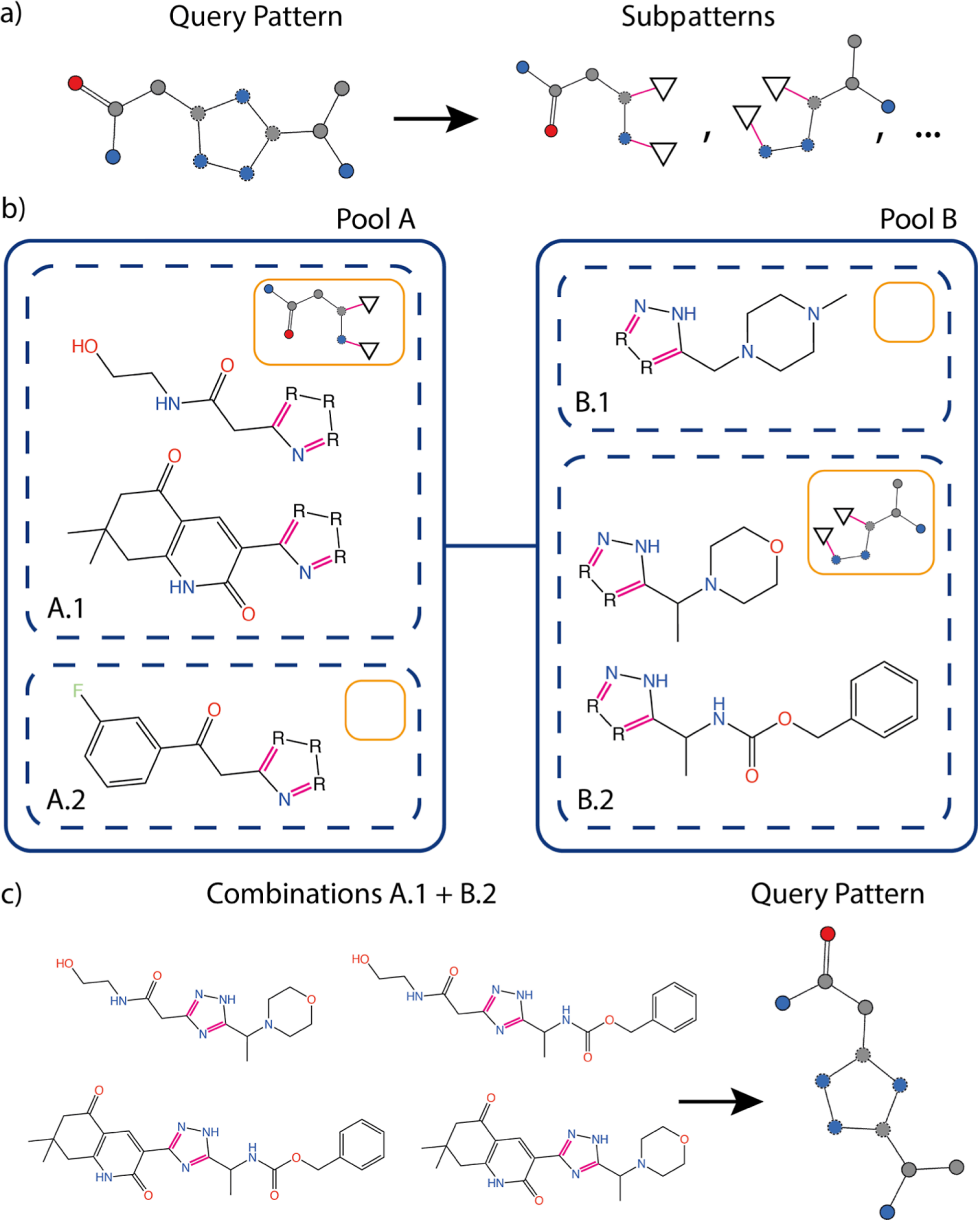
(a) We depict the enumeration of all subpatterns of a query SMARTS pattern. All open bonds are saturated with linker-matching nodes displayed as triangles. The outgoing bonds are marked in pink to emphasize that they have to be matched to the outgoing linker bonds of the fragments. (b) The depicted topological fragment space models a triazole ring closure reaction from the eXplore cookbook^15^ (rxn301). The fragments are grouped by their boundary information, which contains the query subpatterns that match the fragments. (c) Groups A.1 and B.2 contain two subpatterns that together make up the complete query structure. Therefore, all product molecules from the two groups must contain the query structure as a crossing pattern, as demonstrated.

To compute the EPC of a fragment group combination, we build an exemplary product. We then match every query pattern against the exemplary product and investigate all possible atom mappings to determine if it is a crossing pattern. If a mapping is found that contains atoms from more than one fragment, then the query pattern is added to the EPC since it is a crossing pattern. Finally, as the IPCs and EPCs are represented by sets, they can be joined to calculate the resulting distribution. Figure 4 illustrates a simple example of the described process.

Note that the algorithm is not restricted to such simple topologies but computes crossing structures spanning any number of fragments in arbitrary topologies, including macrocycles. For the definition of query patterns, all features of the SMARTS language are supported, except for recursion and disconnected patterns. Additionally, in cyclic topologies, the membership of an atom in a macrocycle cannot be determined at the fragment level. Therefore, the ring membership feature of the SMARTS language works only for fragment internal ring structures or those rings that are formed directly across fragment borders such that ring dummy atoms are included in the fragments.

With the presented definitions, SpaceProp2 produces a distribution that counts how many products in a fragment space contain specific subsets of the query structures. As the number of possible subsets scales exponentially with the number of query patterns, this representation is unsuited for visual inspection. Therefore, we propose additional postprocessing steps to the algorithm that aggregate the information. One such aggregation could combine all product counts of subsets with equal sizes into one entry. The result is a distribution that shows how many products in the fragment space contain none of the patterns, one pattern, two patterns, and so on. This information can be used to determine whether multiple query patterns are contained in compounds at the same time. Alternatively, we can count the occurrence of each query pattern separately to determine which patterns are most common. Both of these aggregated distributions have a clear structure and are easy to interpret. However, the optimal aggregation depends on the use case.

### Optimization

Chemical fragment spaces often use fragments for multiple reactions. When such reactions are translated to a topological fragment space, this results in fragments that appear in multiple topology nodes across multiple topology graphs. During the SpaceProp2 algorithm, each fragment must be processed once to calculate its IPC value and boundary information. For fragments that are reused in multiple topology graphs, this would result in repeated calculation of identical IPC values and boundary information. We, therefore, implemented an additional optimization procedure to speed up the algorithm.

First, after calculating IPC values and boundary information, we associate them with the fragments. Then, before calculating the IPC value and boundary information of a new fragment, we check whether they were already calculated and reuse them to avoid repeated calculations. The lookup is done via unique database keys assigned to each fragment that do not change when a fragment is used in multiple reactions.

In practice, we have found that this technique greatly speeds up the calculation of the molecular pattern property. This is because SMARTS matching itself is a computationally expensive operation. However, for simpler properties such as the TPSA and the rotatable bonds, the additional lookup and storing of the calculated IPC values and boundary information were not faster than the repeated calculation. Therefore, we applied this optimization only to the molecular pattern property.

### Example Molecules

The output of the SpaceProp algorithm is a plain distribution in the form of key-value pairs that show property values associated with their respective product molecule counts. From this result, it is impossible to determine what products in the chemical space are responsible for specific entries in the distribution. To improve the explainability of the results, we added a new feature to SpaceProp2 that lists example molecules for each distribution entry. It is not possible to list all products that make up the product count of a specific property value, as this would be equivalent to enumerating the entire fragment space. Instead, we determine a limited number of example molecules without substantially changing the runtime behavior of the algorithm.

As described above, the first part of the SpaceProp algorithm is the grouping of fragments by their boundary information and the computation of a distribution over the IPCs for each such group. In these distributions, we associate each occurring value with the fragment that produced it. In case multiple fragments share the same IPC we keep only one of them. Later, these IPC distributions are merged to form the final property distributions. During this step, the IPC values of the different distributions are combined with the EPC values of the current fragment group combination. Besides computing the final property value, we additionally group the responsible fragments associated with the IPC values and associate this new fragment combination with the computed property value. Again, if multiple fragment combinations yield the same property value, we keep one of them. Finally, when merging the property distributions of multiple topology graphs, we keep all fragment combinations, storing them in a list if multiple combinations share property values. As a result of this procedure, we associated each property value in the final distribution with at least one fragment combination for each topology graph. From these fragment combinations, we can construct example molecules for each value of interest.

The number of example molecules available for each distribution value is determined by the number of fragment group combinations that yielded equal property values. The runtime and memory consumption of the algorithm are slightly increased due to the additional work of associating the distribution values with the corresponding fragments. However, during the computation of the topology graph distributions, the additional workload is constant for each value in the distribution as every value is associated with only one fragment or fragment combination. Furthermore, during the final merging of the topology graph distributions, the additional workload consists of adding one item to the list of fragment combinations whenever the distributions share the same values and their count values must be added up. Therefore, the increase in the runtime does not substantially change the runtime behavior of the algorithm. However, to obtain the example molecules, the fragments must be combined in a postprocessing step to build full molecules. This step takes time but is separate from the histogram calculation; hence, it does not delay the algorithm itself.

The procedure is deterministic concerning the choice of example products. We always use the first fragment to express a certain IPC value as the corresponding example fragment and process fragments in the order in which they are stored in the database. Therefore, the produced example molecules change only if the internal order inside the database changes. As described above, the number of available example molecules is not known beforehand. To avoid convoluted results, we limit the output in our implementation to 10 example molecules per property value.

## Results

### Fragment Spaces

We applied SpaceProp2 to six different large chemical fragment spaces. The first four are recent versions of the same libraries as used by Bellmann et al.,^12^ namely, Enamine’s REALSpace,^1^ OTAVA’s CHEMriya,^3^ WuXi’s GalaXi,^2^ and the publicly available KnowledgeSpace.^26^ We extend this list by three additional chemical spaces. The first two are eXplore^4^ and Freedom Space,^27^ two commercial fragment spaces developed by eMolecules and ChemSpace in collaboration with BioSolveIT GmbH. The last one is Boehringer Ingelheim’s in-house chemical fragment space BICLAIM.^5^ This space is proprietary and represents the largest collection of compounds considered in this work. Details about the sizes of the different spaces are summarized in Table 1. The given number of fragments refers to the sum of the building blocks contained in all topology nodes in the given fragment space. Since the same building block can be used in multiple reactions, the number of unique fragments is provided to count the deduplicated building blocks that make up the fragment space. For all of the regarded fragment spaces, we used the latest version available in June 2023.

**Table 1:** Sizes of the Chemical Fragment Spaces with Regard to the Number of Encoded Product Molecules, the Number of Fragments, and the Number of Unique Fragments

space	prod.	frag.	u. frag.
Freedom Space	1.8 × 10^8^	7.0 × 10^4^	3.5 × 10^4^
GalaXi	1.2 × 10^10^	1.7 × 10^5^	2.0 × 10^4^
CHEMriya	2.2 × 10^10^	1.9 × 10^5^	2.8 × 10^4^
REALSpace	6.5 × 10^10^	2.3 × 10^6^	2.4 × 10^5^
eXplore	1.4 × 10^12^	1.9 × 10^6^	2.9 × 10^5^
KnowledgeSpace	2.9 × 10^14^	6.6 × 10^5^	3.2 × 10^5^
BICLAIM	3.4 × 10^17^	3.9 × 10^7^	2.5 × 10^5^

### Validation

To validate the new property histogram calculations, we used the two subspaces of KnowledgeSpace that were also used by Bellmann et al.^12^ for validation. With approximately 790,000 and 1,100,000 molecules, these collections are small enough for enumeration. We manually computed the new property distributions for the enumerated subspaces and ensured that the results matched the SpaceProp2 output. This approach is identical with the workflow used by Bellmann et al.

### Molecular Patterns

To demonstrate the calculation of molecular pattern distributions with SpaceProp2, we chose covalent drug discovery as an application scenario. In contrast to conventional drugs, these drugs form covalent bonds with their protein targets, which can offer more stable and often irreversible protein–ligand complexes. They have been in use for more than 100 years, the most prominent example being acetylsalicylic acid.^28^ The covalent bonds are formed by specific reactive groups. This mechanism of action was historically often discouraged as potential binding to unintended targets may lead to unwanted side effects. However, recent developments have shown that the rational design of covalently binding drugs can lead to highly potent and selective drugs, even for previously thought undruggable targets.^29^ A recent review by Péczka et al.^30^ introduced an extensive collection of potential reactive groups used in the literature for covalent drug discovery, termed electrophilic warheads. From the 121 molecular structures curated in the respective WHdb, we extracted 141 SMARTS patterns. Details of this process are provided in the Supporting Information. We used the 141 SMARTS patterns as a query for SpaceProp2 to determine whether the regarded chemical fragment spaces contain products with potential electrophilic warheads.

### Runtime

The following factors determine the runtime of SpaceProp2:

1. The number of fragments in a fragment space
2. The complexity of the operations done on each fragment to calculate the IPCs
3. The degree to which fragments can be grouped by their IPCs
4. The range of different IPC values that the fragments assume

The dependence on the number of fragments and computation of the IPCs stems from the fact that each fragment in a fragment space must be processed once. The degree of grouping of fragments by their IPCs influences the runtime because each combination of groups must be processed once to compute the shared EPC. Finally, the IPC value range determines the number of different entries in the IPC distributions of the fragment groups. When we combine these, each combination of IPC values forms a new entry in the final distribution. The number of such combinations increases significantly with the number of different IPC values that the fragments assume. For the molecular pattern property, the IPC value range scales with the number of query patterns. This follows from the fact that the IPCs are represented as subsets of patterns that are contained in the fragments. Therefore, the IPC value range is represented by the number of possible subsets of the query patterns, which increases with the number of query patterns.

Table 2 displays the runtimes of the SpaceProp2 algorithm on the different fragment spaces. The measurements for all fragment spaces except BICLAIM were taken on a standard computer with 16 GB of RAM and 2.6 GHz processors. The program was set to run on six cores in parallel. The BICLAIM calculations were done on 24 cores on a computing cluster machine with 80 GB of RAM and 1.1 GHz processors. The precision for the TPSA calculation was set to two decimal places, so no rounding was applied. The query patterns were preprocessed in a separate step and reused for all fragment spaces, as described above. The preprocessing of the query patterns took 10 min and 24 s on the described standard computer.

**Table 2:** SpaceProp2 Runtimes in hours:minutes:seconds

space	TPSA	Rot. B.	SMARTS^a^
Freedom Space	00:00:06	00:00:06	00:03:00
GalaXi	00:00:13	00:00:13	00:02:34
CHEMriya	00:00:17	00:00:17	00:01:45
REALSpace	00:03:19	00:03:20	00:10:48
eXplore	00:02:38	00:02:39	00:17:32
KnowledgeSpace	00:01:05	00:01:05	00:15:24
BICLAIM	00:59:54	01:00:49	259:52:34

a The runtimes for the SMARTS distribution calculation do not include the time for the query pattern preprocessing (00:10:24).

The data clearly show that a more complex operation like the SMARTS matching leads to substantially longer runtimes compared to simpler operations such as the calculation of the TPSA. It also suggests that the runtime for the TPSA and rotatable bonds directly scales with the number of fragments contained in the fragment spaces. Whereas, the runtime for the molecular pattern calculation correlates with the number of unique fragments rather than the total number of fragments. This is likely due to the optimization step that reuses already calculated IPC values, so each unique fragment is computed only once. Finally, the deviations from the correlation between runtime and unique fragment count for the molecular patterns property are likely caused by the different grouping degrees of the fragments and the different IPC value ranges, which depend highly on the abundance of the query patterns in a particular fragment space.

### Distributions

#### TPSA and Rotatable Bonds

**Figure 5:**
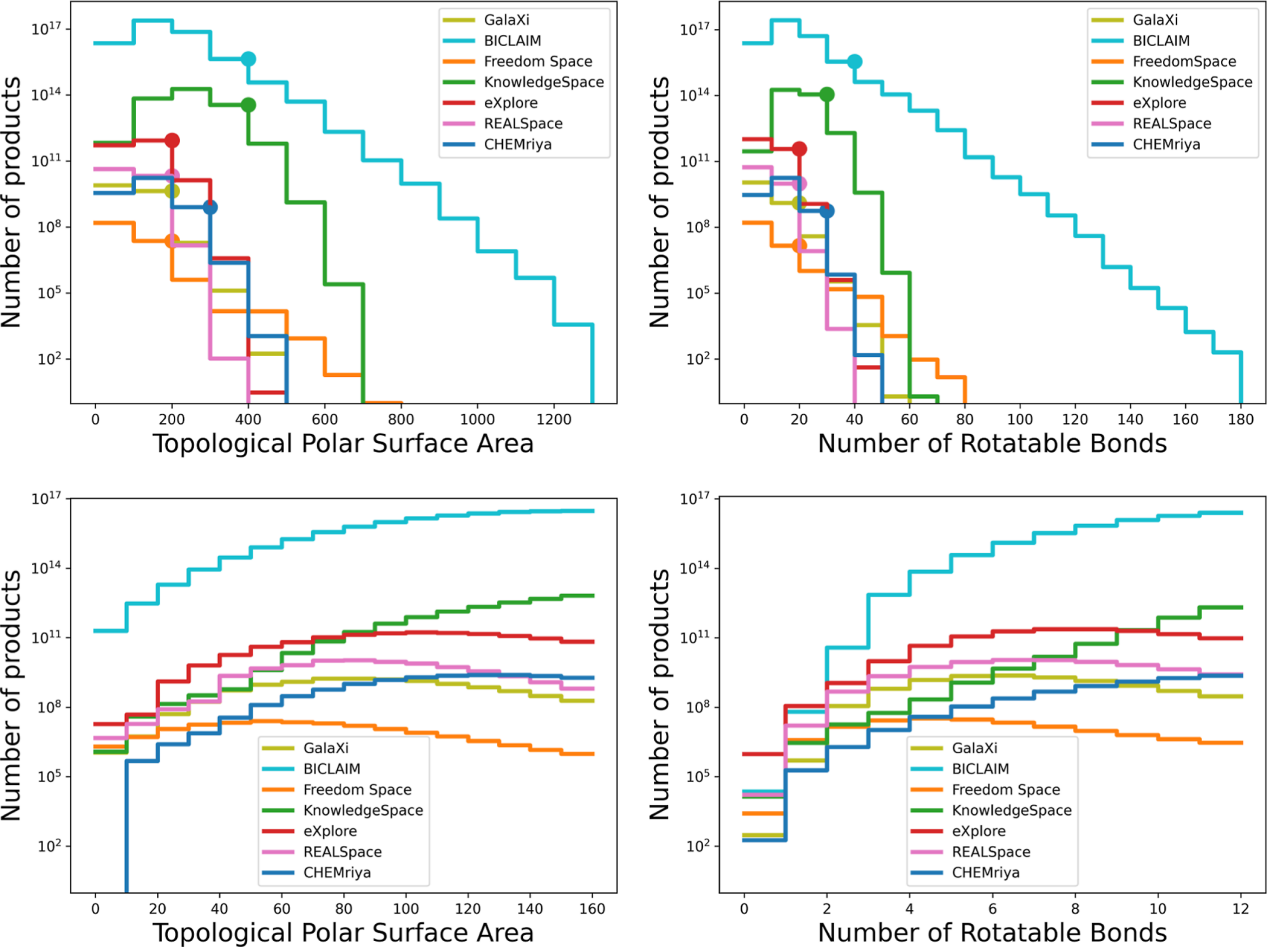
Four diagrams show the distribution of the TPSA (left) and number of rotatable bonds (right) for all considered fragment spaces on a logarithmic scale. The two diagrams on the top show the full value ranges for both properties. For a better interpretation of the logarithmic scale, the dot on each line indicates the 99% mark, meaning that less than 1% of products lie to the right of the marked value. The two diagrams on the bottom show the distributions within the value ranges proposed by Veber et al.^21^ in more detail.

**Figure 6:**
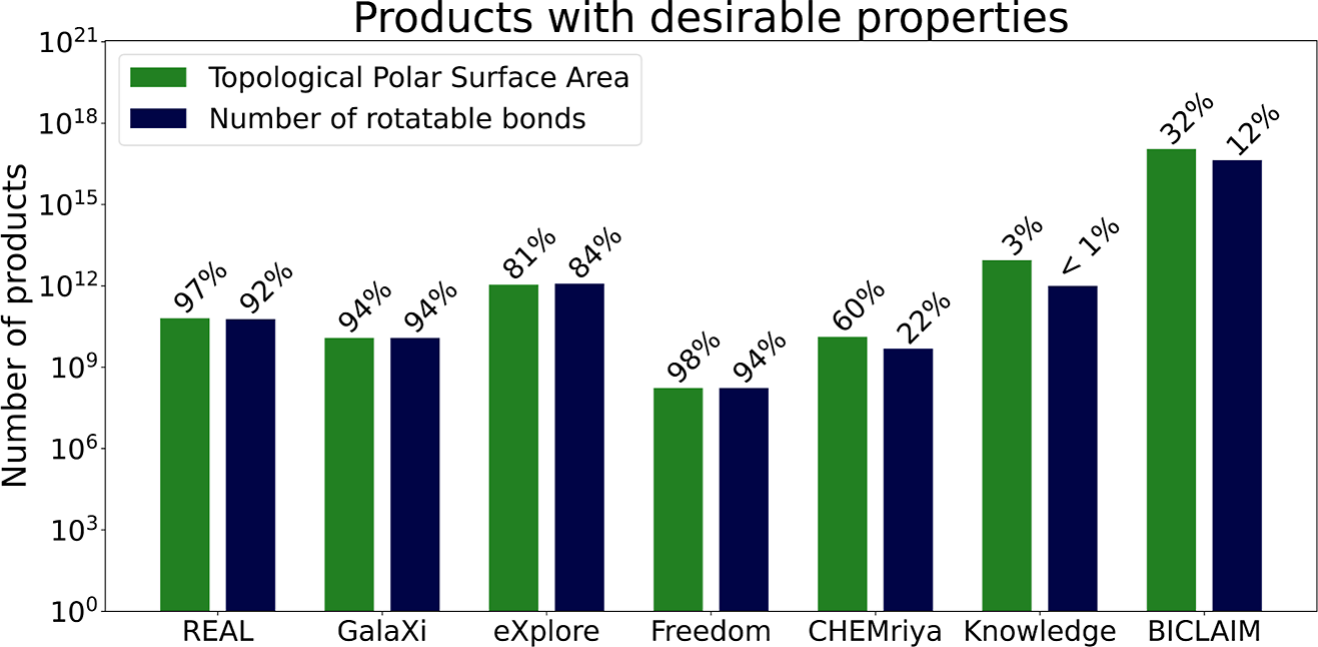
Comparison of the fragment spaces with regard to the number of molecules within the thresholds for potential oral bioavailability of compounds as proposed by Veber et al.^21^ The bars display the absolute number of products on a logarithmic scale while the given percentages show the relative number of products with regard to the total sizes of the fragment spaces.

Figures 5 and 6 summarize the results for the TPSA and rotatable bonds calculation. From Figure 5, it is evident that there are significant differences in the TPSA and rotatable bond distributions among the fragment spaces. As an example, we see that BICLAIM as the largest of the regarded fragment spaces contains a higher percentage of molecules with larger TPSA and more rotatable bonds than the compounds in the other spaces. However, the size of a fragment space does not directly determine the breadth of its distribution. This is demonstrated by Freedom Space, which, despite being the smallest of the regarded spaces, contains molecules with larger TPSA values and more rotatable bonds than all other spaces except BICLAIM. Another detail that stands out is the TPSA distribution of CHEMriya which shows that the entire library does not contain any molecules with TPSA below 10.

Veber et al.^21^ proposed that a potentially bioavailable compound should have a maximum TPSA of 140 Å^2^ and a maximum of 10 rotatable bonds. As shown in Figure 6, high percentages of compounds in REALSpace, GalaXi, and Freedom Space fulfill these criteria for TPSA and rotatable bonds, respectively. For the other spaces, this applies to significantly smaller portions, KnowledgeSpace being the most extreme example with only 3% and less than 1% of compounds fulfilling the criteria. It is important to note, however, that these relative numbers do not reflect the absolute numbers due to the enormous size differences between the regarded fragment spaces. Therefore, BICLAIM, as an example, still contains orders of magnitude more compounds fulfilling the criteria than any of the other spaces despite its comparably low percentages of 32% and 12%.

#### Molecular Patterns

As discussed above, the SMARTS distributions calculated by SpaceProp2 count how many products in a chemical fragment space contain any subset of the provided query patterns. This information can be aggregated in different ways. In our chosen application example, we focus on evaluating if the regarded fragment spaces contain products with any one of the provided query structures. To this end, we sum up the product counts of all query structure subsets with at least one structure. Table 3 shows the total number and relative frequency of compounds for each fragment space containing at least one of the potential electrophilic warheads. The results indicate that although none of the regarded fragment spaces were built for covalent drug discovery, all of them contain a significant amount of potential candidate compounds for this purpose. Correlating with the total size of the fragment spaces, most such compounds are contained in BICLAIM, KnowledgeSpace, and eXplore.

**Table 3:** Number of Products Containing at Least One Potential Electrophilic Warhead

space	# products	% products
Freedom Space	4.0 × 10^7^	22.7
GalaXi	2.1 × 10^9^	17.2
CHEMriya	4.6 × 10^9^	21.6
REALSpace	9.7 × 10^9^	15.0
eXplore	4.1 × 10^11^	29.3
KnowledgeSpace	1.7 × 10^14^	57.6
BICLAIM	1.1 × 10^17^	31.9

**Figure 7:**
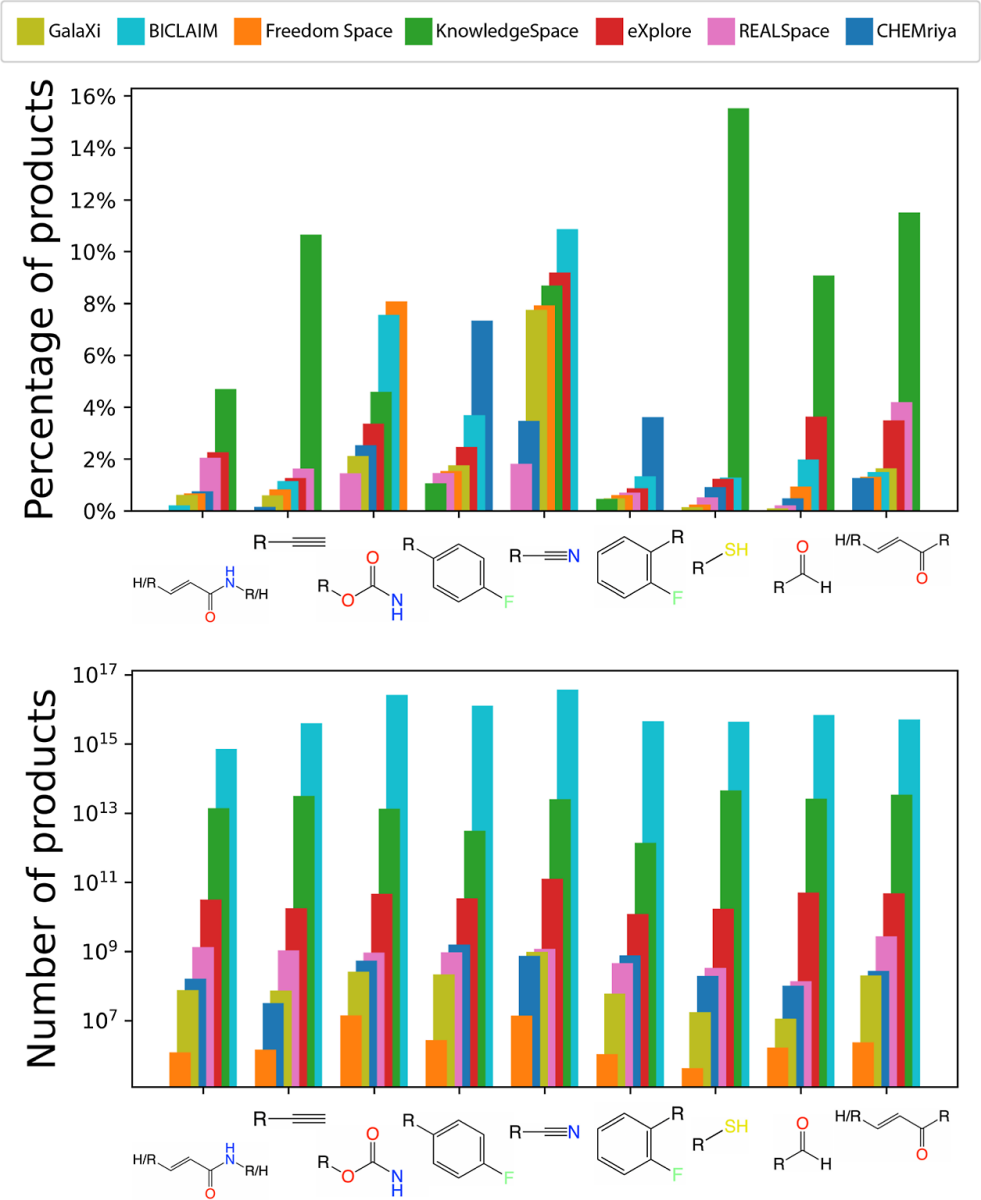
The most commonly occurring potential electrophilic warhead structures in the regarded fragment spaces, presented in relative (top) and absolute counts (bottom).

**Figure 8:**
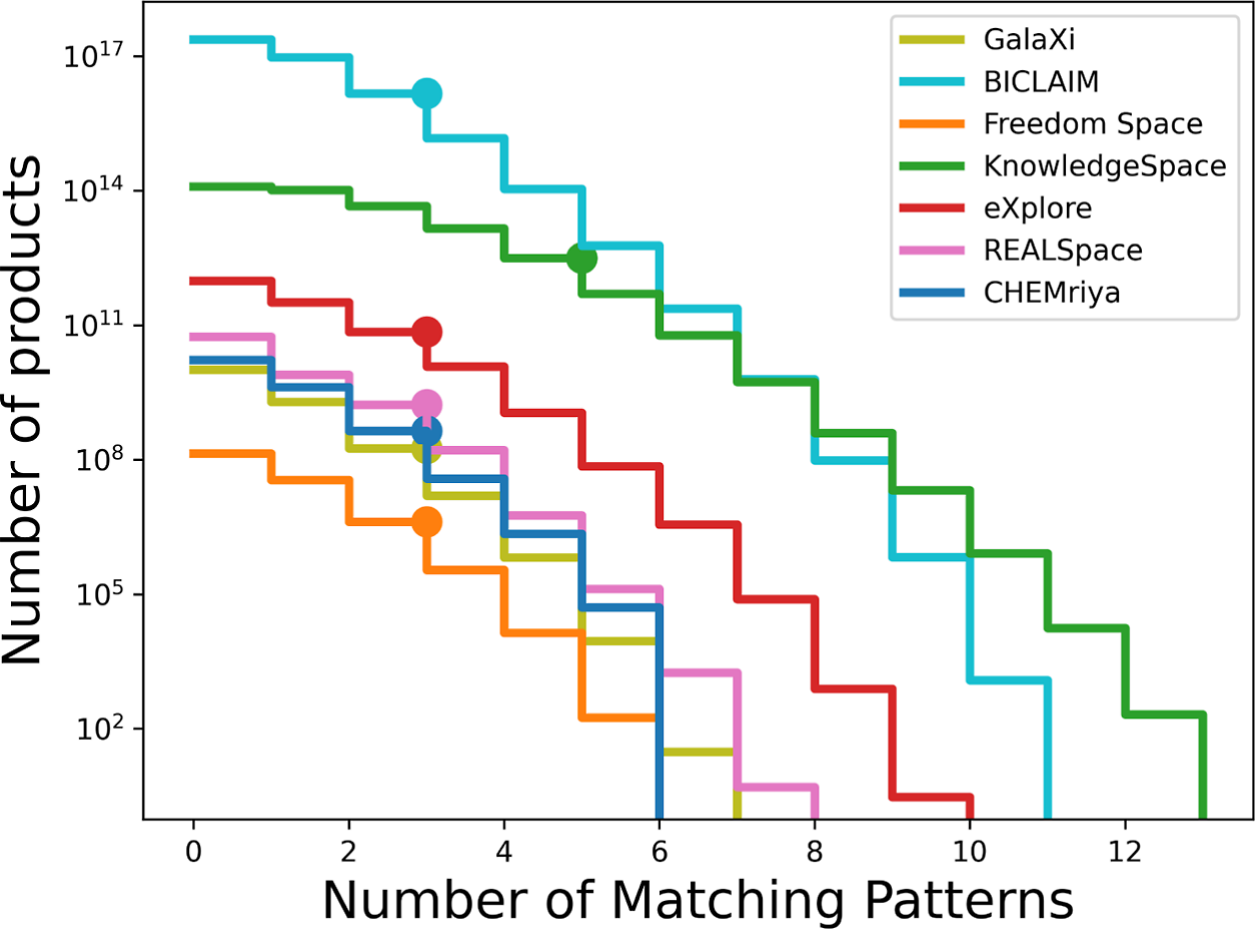
Histogram counting how many products of the regarded fragment spaces contain none, one, or multiple query patterns. Product counts are given on a logarithmic scale. The dot on each line again indicates the 99% mark, such that a maximum of 1% of compounds in a fragment space contain more than the marked number of query patterns.

Figures 7 and 8 show different aggregations of the output produced by SpaceProp2. We use the aggregation by query pattern to find out which of the query patterns occur in which numbers. We determined the set of the most common patterns by joining the three most frequently occurring query structures in each of the fragment spaces. The result is a set of 9 structures. Figure 7 shows the relative and absolute occurrences of these nine most common patterns. The relative occurrences in the top diagram, in particular, show the significant differences in the structural composition of the spaces. KnowledgeSpace, for example, contains many of the query patterns in comparably high percentages of up to 15% while REALSpace contains none of the patterns in more than 5% of products. CHEMriya, as another example, has the highest share of compounds containing *p*- and *o*-fluorobenzene structures but has a lower frequency of nitrile structures compared to all other spaces except REALSpace. Again, these differences in relative occurrence are mitigated by the size differences between the spaces as the absolute numbers in the bottom diagram of Figure 7 shows. It is important to note that the molecular patterns can contain each other. An example of this fact is the pattern pair for α,β-unsaturated ketones (rightmost structure) and α,β-unsaturated amides (leftmost structure). The ketone pattern is contained in the amide pattern such that every α,β-unsaturated amide is also regarded as an α,β-unsaturated ketone. The relative and absolute counts of the ketones in Figure 7, therefore, also include the counts of the amides.

**Figure 9:**
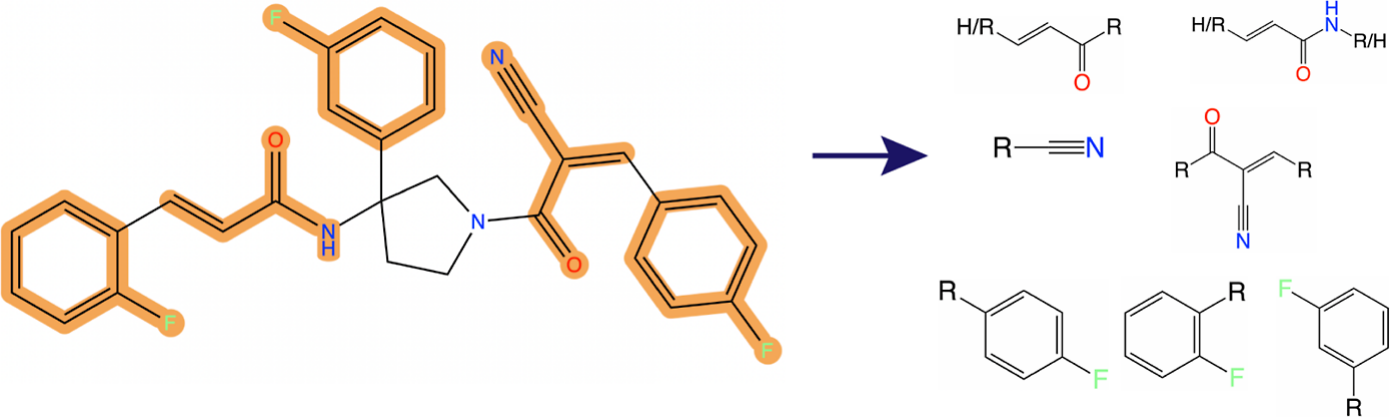
Example molecule from REALSpace that contains seven potential electrophilic warhead structures. The structures are shown on the right and their occurrence is highlighted in the example molecule.

In Figure 8, we see that all regarded fragment spaces contain molecules with multiple potential electrophilic warheads. Visualized by the dot marks in the diagram, we see that for all fragment spaces except for KnowledgeSpace, 99% of the product molecules contain either none, one, or two of the query patterns. However, some compounds match many of the query patterns. To analyze one of these rare cases, we used the new example molecule feature to extract an example molecule from REALSpace that contains seven of the query patterns. It is shown in Figure 9. When inspecting the contained query patterns, we can see that three of the contained patterns are different configurations of fluorobenzene structures. Additionally, the molecule contains an α,β-unsaturated amide structure which includes an α,β-unsaturated ketone, as described above. Finally, the cyanoenone pattern contains a nitrile structure, which is itself part of the query patterns. Depending on the interpretation, it is, therefore, possible to argue that the example molecule contains only three different electrophilic warhead patterns: fluorobenzene (in three different configurations), an α,β-unsaturated amide, and a cyanoenone. This result highlights the importance of the introduced explainability feature, as inspecting example molecules for particular histogram values can ensure the correct interpretation of the SpaceProp2 results.

### Follow-Up Analysis

**Figure 10:**
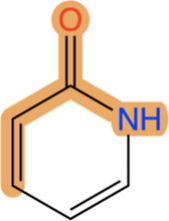
α,β-unsaturated amide pattern matching in a lactam structure.

The presented results revealed that α,β-unsaturated amides are among the most common patterns. We used the SMARTS expression
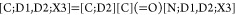
to describe these amides. Note that this expression does not include any information about ring membership and would, therefore, also match lactam structures as in Figure 10.

Because such lactam structures show a different electrophilic potential, we performed a follow-up analysis to investigate how many of the previous matches found for α,β-unsaturated amides are contained in ring structures. We ran SpaceProp2 on REALSpace with two modified SMARTS expressions. In the first expression, we specified that the carbonyl carbon must be part of a ring structure. In the second expression, we specified that the same atom cannot be part of a ring. The results are shown in Table 4.

**Table 4:** Occurrence Rates of the Original, Cyclic, and Noncyclic α,β-Unsaturated Amide Patterns

pattern	# products
original	1,325,043,741
noncyclic	1,324,977,576
cyclic	66,169

# contained patterns	# products
0	63,612,473,590
1	1,325,043,737
2	4

**Figure 11:**
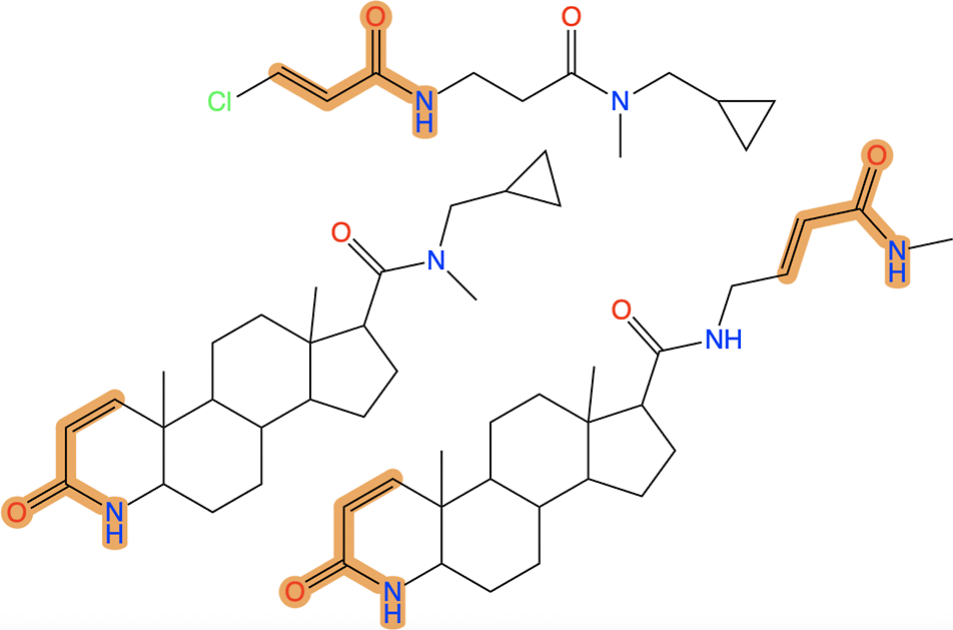
Example compounds from REALSpace containing cyclic and noncyclic α,β-unsaturated amide structures as well as both at the same time.

The data show that only a tiny fraction of the found α,β-unsaturated amide structures correspond to cyclic pattern matches in REALSpace. Note that the sum of the product counts for the ring and nonring patterns surpasses the product count for the original pattern by four. This difference is explained by the fact, that REALSpace contains four compounds that match both of the modified patterns in separate locations. Figure 11 displays example compounds for the different groups, as provided by the new example molecule feature.

The SpaceProp2 calculation for the modified patterns took 200 s to complete. Note that this is significantly shorter than the duration of the SpaceProp2 run with all of the warhead structures, which demonstrates the impact of the number of query patterns on the algorithm’s runtime.

## Conclusions

Due to the immense size and the combinatorial nature of chemical fragment spaces, sophisticated analysis methods are required to anticipate the molecular properties of the products contained. Traditional methods to calculate property distributions rely on enumeration, which is impossible for large fragment spaces. The SpaceProp algorithm computes exact property distributions for large fragment spaces without enumerating them, providing results in reasonable runtimes. In this work, we present SpaceProp2, an extended version of the SpaceProp algorithm that enables the additional calculation of molecular property distributions for TPSA, the number of rotatable bonds, and the occurrence of SMARTS patterns.

The distributions of TPSA and the number of rotatable bonds produced by SpaceProp2 offer further insights into the drug-likeness of compounds in a chemical fragment space. The molecular pattern distributions offer analyses concerning project-specific structural features. The new feature allows the efficient, parallel matching of multiple SMARTS patterns in large chemical fragment spaces, which was not possible before. The newly created opportunity to extract sample molecules for certain feature value bins enables the interpretation of chemical space property distributions for the first time.

All in all, SpaceProp2 is a powerful analysis tool for large chemical fragment spaces with two major applications. First, it can aid researchers in finding the most suitable search library for a project. Second, it can aid in the design process of chemical fragment spaces. The produced property distributions can reveal undesired contents and potential gaps in the structural diversity of a fragment space. With the help of the example molecules the algorithm provides, users can identify and remove fragments that lead to unwanted properties. On the other hand, new fragments and reactions can be added with their impact on the fragment space directly visible in the property distributions. With future research, we believe that this approach can be further refined to build optimized chemical fragment spaces that enrich compounds with user-defined properties.

## Data Availability

SpaceProp2 will be available for Linux, MacOS, and Windows as part of the NAOMI ChemBio Suite at https://uhh.de/naomi. It will be free for academic use and evaluation purposes. KnowledgeSpace in its topological fragment space representation can be accessed at https://www.zbh.uni-hamburg.de/forschung/amd/datasets.html. For a detailed description of the chemical spaces used in this publication, see https://www.biosolveit.de/infiniSee. Enamine’s REAL Space, WuXi’s GalaXi Space, OTAVA’s CHEMriya Space, and Chemspace’s Freedom Space cover commercially available on-demand molecules. eMolecule’s eXplore Space covers fragment combinations in conjunction with robust reactions. All spaces can be obtained from the respective compound providers or Bio-SolveIT GmbH.

## Supporting Information Available

Details about the distinction between fragment-additive and nonfragment-additive properties and a detailed list of the electrophilic warheads SMARTS patterns used in this work (PDF)
