# Supplementary material for: Enhanced Calculation of Property Distributions in Chemical Fragment Spaces
Source: J Chem Inf Model. 2024 Mar 11;64(6):2008–20. doi: 10.1021/acs.jcim.4c00147 (PMC10966640; doi:10.1021/acs.jcim.4c00147)
Supplement: Supplementary file 1 — ci4c00147_si_001.pdf [file ci4c00147_si_001.pdf]

Supporting Information: Enhanced Calculation of Property Distributions in Chemical Fragment Spaces

Justin Lübbers,[*,†] Uta Lessel,[*,‡] and Matthias Rarey[*,†]

†*Universität Hamburg, ZBH - Center for Bioinformatics, Research Group for Computational Molecular Design, 22761 Hamburg, Germany*

‡*Computational Chemistry, Boehringer Ingelheim Pharma GmbH & Co. KG, 88437 Biberach an der Riss, Germany*

E-mail: justin.luebbers@uni-hamburg.de; uta.lessel@boehringer-ingelheim.com; matthias.rarey@uni-hamburg.de

Fragment-additive properties

In this work, we do not differentiate between fragment-additive and nonfragment-additive properties. A fragment-additive property as defined by Bellmann et al. is characterized by the fact that the property value of a fragment does not depend on any information about the connected fragments. Therefore, the property value of a product solely consists of the combination of IPCs of the contained fragments. Bellmann et al. proposed a simpler version of the SpaceProp algorithm for fragment-additive properties that skips grouping the fragments of a topology node based on their boundary information. Instead, all fragments of a topology node are grouped together in one group.

The same procedure can be achieved by defining the boundary information as empty for

all fragments for any fragment-additive property. When applying the SpaceProp algorithm for nonfragment-additive properties, all fragments of a topology node then have identical boundary information and are grouped together in one group. The resulting procedure is equivalent to the SpaceProp algorithm for fragment-additive properties. For simplicity, we, therefore, consider the general SpaceProp algorithm to follow the proposed strategy for nonfragment-additive properties and define all boundary information as empty for any fragment-additive property.

Electrophilic Warheads SMARTS Patterns

We extracted 141 SMARTS patterns from the WHdb created by Péczka et al.. Not all 125 contained structures were suitable for our purpose, while some structures resulted in multiple SMARTS patterns. Generally, as SpaceProp2 only supports clearly defined, non-recursive SMARTS patterns, we excluded structures that contain unspecified alkyl, peptityl, aryl, heteroaryl, heterocyclic, LG or EWG groups. Additionally, because our application scenario focuses on electrophilic warheads that are part of a molecule, we excluded all structures without an explicit R group. In structures with only one R/H group, the R/H group was treated as an R group. For all atoms, we allowed only the depicted outgoing bonds. Therefore, atoms without an R group were assumed to be saturated with hydrogen atoms. These are the resulting 141 SMARTS patterns, named by their appearance in the original WHdb[2]:

2H-azirine:

N1[C;D2][C;D3]=1

3-oxo-beta-sultam:

[N;D2,D3]1C(=O)[C;D4]S1(=O)=O

5-methylene pyrrolone:

[C;D1]=C1[C;D2]=[C;D2]C(=O)[N;D3]1

α,β-unsaturated aldehyde (acrylic aldehyde):

[C;D2;X3]=[C;D2][C;D2]=O

α,β-unsaturated amide (acrylamide):

[C;D1,D2;X3]=[C;D2][C](=O)[N;D1,D2;X3]

α,β-unsaturated cyanoamide (cyanoacrylamide):

[C;D1,D2;X3]=C([C;D2][N;D1])C(=O)[N;D1,D2;X3]

α,β-unsaturated ester (acrylate, fumarate):

[C;D1,D2;X3]=[C;D2]C(=O)[O;D2]

α,β-unsaturated fluoroamide (fluoroacrylamide):

[C;D1,D2;X3]=C(F)C(=O)[N;D1,D2;X3]

α,β-unsaturated ketone:

[C;D1,D2;X3]=[C;D2][C;D3]=O

α,β-unsaturated nitrile (acrylonitrile):

[C;D2;X3]=[C;D2]C#N

α,β-unsaturated sulfonamide:

[C;D1,D2;X3]=[C;D2]S(=O)(=O)[N;D1,D2;X3]

α,β-unsaturated sulfone:

[C;D1,D2;X3]=[C;D2][S;D4](=O)(=O)

Acyl hydroxamate:

O=[C;D3][N;D2]O[C;D3]=O

α-ketoaldehyde (glyoxyl):

O=[C;D3][C;D2]=O

α-ketoamide:

O=[C;D3]C(=O)[N;D1,D2;X3]

Aldehyde:

[C;D2;X3]=O

Alkene (difluoro):

[C;D2;X3]=C(F)F

Alkene (nitro):

[C;D2;X3]=[C;D2]N(=O)=O

[C;D2;X3]=[C;D2][N;+](=O)[O;D1;-]

Alkyne (terminal):

[C;D2]#[C;D1]

Alleneamides:

[C;D1]=C=[C;D2]C(=O)[N;D2;X3]

Aza-peptides:

O=[C;D3][N;D2][N;D3]C(=O)[O;D2]

Aziridine:

[N;D2]1[C;D2][C;D3;X4]1

Azodicarboxamides:

[Ac;D1][N;D2][N;D3]C(=O)N=NC(=O)[N;D2;X3]

Benzoxatiazinone:

O=C1Oc2[c;D2][c;D2][c;D2][c;D2]c2S(=O)(=O)[N;D3]1

Benzoxazin-4-one:

O=C1O[C;D3]=Nc2[c;D2][c;D2][c;D2][c;D2]c12

β-lactam:

[N;D2,D3]1C(=O)[C;D4][C;D4]1

β-lactone (mono and bicyclic):

O1C(=O)[C;D4][C;D4]1

Boronic acid:

[B;D3]([O;D1])[O;D1]

Boronic acid carbonyl:

O=[C;D3]c1[c;D2][c;D2][c;D2][c;D2]c1B([O;D1])[O;D1]

Bromo/chlorodihydroisoxazole:

[Br,Cl]C1[C;D2][C;D3;X4]ON=1

β-sultame:

[N;D2,D3]1[C;D4][C;D4]S1(=O)=O

Carbamate:

[O;D2]C(=O)[N;D2;X3]

Cyanamide:

[N;D2,D3;X3]C#N

Cyanoenone:

O=[C;D3]C(C#N)=[C;D2;X3]

Cyclobutenaminone:

O=[C;D3][N;D3]C1C([C;D1])([C;D1])C(=O)C=1[H,Br]

Cyclopropene:

[C;D2,D3]1=[C;D2,D3][C;D2,D3;X3]1

Cyclopropenone:

[C;D3]1=[C;D2]C1=O

Diacyl furoxane (masked nitrile oxide):

O=[C;D3]C1=NO[N;+]([O;D1;-])=C1[C;D3]=O

Diazaborine:

[O;D1][B]1c2[c;D2][c;D2][c;D2][c;D2]c2[C;D3]=[N;D2][N;D3]1

Diazocarboxamide:

[N;D1;-]=[N;D2;+]=[C;D3]C(=O)[N;D2;X3]

[N;D1;-]=[N;D2][C;D3;+]C(=O)[N;D2;X3]

[N;D1]#[N;D2;+][C;D3;-]C(=O)[N;D2;X3]

[N;D1;+]=[N;D2][C;D3;-]C(=O)[N;D2;X3]

Diazocarbocamide (cyclic):

O=C1N=NC(=O)[N;D3]1

Diazomethyl ketone:

O=[C;D3][C;D2]=[N;D2;+]=[N;D1;-]

O=[C;D3][C;D2;+][N;D2]=[N;D1;-]

O=[C;D3][C;D2;-][N;D2;+]#[N;D1]

O=[C;D3][C;D2;-][N;D2]=[N;D1;+]

Disulfide:

[S;D2;v2][S;D2;v2]

Enol-cyclocarbamate:

O=[C;D3][C;D2]=C1OC(=O)N2[C;D2][C;D2][C;D2][C;D3]12

Epoxide (spiro):

O1[C;D3;X4]C12[C;D2][C;D2][C;D3;X4][C;D2][C;D3;X4]2

Epoxide, epoxyketone, epoxysuccinate:

O1[C;D2][C;D3]1[C;D3]=O

O1[C;D2][C;D3]1C(=O)[O;D2]

O1[C;D3]([C;D3]=O)[C;D3]1[C;D3]=O

O1[C;D3]([C;D3]=O)[C;D3]1C(=O)[O;D2]

O1[C;D3](C(=O)[O;D2])[C;D3]1C(=O)[O;D2]

Ester (activated: succinimidyl, acyl phosphate, nitrophenyl, dibromophenyl, carboxyphenyl):

O=[C;D3]ON1C(=O)[C;D2][C;D2]C(=O)1

O=[C;D3]OP(=O)([O;D2])[O;D2]

Ethynylphosphonamidate:

[N;D2]P(=O)([O;D1])C#[C;D1]

Fluorobenzene, perfluorobenzene:

[c;D3]1c(F)[c;D2][c;D2][c;D2][c;D2]1

[c;D3]1[c;D2]c(F)[c;D2][c;D2][c;D2]1

[c;D3]1[c;D2][c;D2]c(F)[c;D2][c;D2]1

[c;D3]1c(F)c(F)[c;D2][c;D2][c;D2]1

[c;D3]1c(F)[c;D2]c(F)[c;D2][c;D2]1

[c;D3]1c(F)[c;D2][c;D2]c(F)[c;D2]1

[c;D3]1c(F)[c;D2][c;D2][c;D2]c(F)1

[c;D3]1[c;D2]c(F)c(F)[c;D2][c;D2]1

[c;D3]1[c;D2]c(F)[c;D2]c(F)[c;D2]1

[c;D3]1c(F)c(F)c(F)[c;D2][c;D2]1

[c;D3]1c(F)c(F)[c;D2]c(F)[c;D2]1

[c;D3]1c(F)c(F)[c;D2][c;D2]c(F)1

[c;D3]1c(F)[c;D2]c(F)c(F)[c;D2]1

[c;D3]1c(F)[c;D2]c(F)[c;D2]c(F)1

[c;D3]1[c;D2]c(F)c(F)c(F)[c;D2]1

[c;D3]1[c;D2]c(F)c(F)c(F)c(F)1

[c;D3]1c(F)[c;D2]c(F)c(F)c(F)1

[c;D3]1c(F)c(F)[c;D2]c(F)c(F)1

[c;D3]1c(F)c(F)c(F)c(F)c(F)1

Fluorosialyl fluoride:

C(=O)([O;D2])C1(F)O[C;D3;X4][C;D3;X4][C;D3]([O;D2])[C;D3]1(F)

Fluorosulfate:

[O;D2]S(=O)(=O)F

Furane:

[C;D3]1=[C;D3]O[C;D2]=[C;D2]1

γ-lactame, γ-lactone, γ-sultone:

O=C1[C;D3;X4][C;D3;X4][C;D3;X4]O1

O=C1[C;D3;X4][C;D3;X4][C;D3;X4][N;D2,D3]1

O=C1[C;D3;X4][C;D3;X4][C;D3;X4]S1(=O)=O

Haloacetamide, dihaloacetamide:

[N;D2;X3]C(=O)C([F,Cl,Br,I])([F,Cl,Br,I,H])[H]

Haloacetamidine:

[N;D2;X3]C(=[N;D1])[C;D2][F,Cl,Br,I]

Halomethyl ketone:

O=[C;D3][C;D2][F,Cl,Br,I]

Hydrazone:

[N;D2;X3]N=[C;D2;X3]

Imine (Mannich reaction between aldehyde, amine and Tyr aromatic ring):

[N;D2]=[C;D2]

Iminosulfoxy difluoride:

[N;D2]=S(F)(F)=O

Isocoumarin:

[O;D2]C1=C(Cl)c2[c;D2][c;D2][c;D2][c;D2]c2C(=O)O1

Isocyanate:

[N;D2]=C=O

Isothiocyanate:

[N;D2]=C=[S;D1]

Isoxazolidinone:

O=C1[C;D3;X4][C;D2]O[N;D3]1

Isoxazolium:

[N;D3;+]1=[C;D2][C;D3]=[C;D3]O1

Ketone (activated: acyloxymethyl, 2-boronyl-phenyl, trifluoromethyl, heterocyclic):

[C;D2;X4][C;D2]C(=O)C(F)(F)F

Maleimide:

O=C1[C;D2]=[C;D2]C(=O)[N;D3]1

Methanethiosulfonate:

[S;D2;v2]S(=O)(=O)[C;D1]

N-carbamoyl pyridinium salt:

[O;D2]C(=O)N([C;D1])[n;+]1[c;D3][c;D2][c;D2][c;D2][c;D2]1

[O;D2]C(=O)N([C;D1])[n;+]1[c;D2][c;D3][c;D2][c;D2][c;D2]1

[O;D2]C(=O)N([C;D1])[n;+]1[c;D2][c;D2][c;D3][c;D2][c;D2]1

Nitrile imine and precursors e.g tetrazole, hydrazonoyl halogenide:

[N;D3]1N=N[C;D3]=N1

[N;D2;X3]N=[C;D3][F,Cl,Br,I]

Nitrile, cyanoacetamide:

[C;D2]#N

Nitroalkyl (nitropropionate, masked electrophile):

[O;D2]C(=O)[C;D2][C;D2]N(=O)=O

[O;D2]C(=O)[C;D2][C;D2][N;+](=O)[O;D1;-]

Nitroisoxazoles, α-nitroketoximes and nitrolic acids (masked nitrile oxide):

N(=O)(=O)C1=[C;D3]ON=[C;D3]1

[N;+](=O)([O;D1;-])C1=[C;D3]ON=[C;D3]1

[O;D1]N=[C;D3][C;D2]N(=O)=O

[O;D1]N=[C;D3][C;D2][N;+](=O)[O;D1;-]

[O;D1]N=[C;D3]N(=O)=O

[O;D1]N=[C;D3][N;+](=O)[O;D1;-]

N-oxyl radical:

[O;D1;H0;+0]N1[C;D3]2[C;D2][C;D3;X4][C;D2][C;D3]1[C;D2][C;D2][C;D2]2

Oxaziridine:

[C;D3;X4]1O[N;D3]1

Phosphonate:

[P;D4](=O)([O;D1,D2])[O;D1,D2]

Phosphonyl fluoride:

[P;D4](=O)(F)[O;D1,D2]

Propiolamide, propiolate. propynone:

[C;D1,D2]#CC(=O)[O;D2]

[C;D1,D2]#CC(=O)[N;D1,D2;X3]

Propiolonitrile:

[C;D2]#CC#N

PTAD:

O=C1N=NC(=O)[N;D3]1

Quinone, naphthoquinone:

O=C1[C;D2,D3]=[C;D2,D3]C(=O)[C;D2]=[C;D2]1

O=C1[C;D2]=[C;D2,D3][C;D2,D3]=[C;D2]C(=O)1

Saccharin and derivatives:

O=C1[N;D3]S(=O)(=O)c2[c;D2][c;D2][c;D2][c;D2]c12

Se-N bond, 1,2-benzisoselenazol-3(2H)-one:

O=C1[N;D3][Se;D2]c2[c;D2][c;D2][c;D2][c;D2]c12

S-N bond, 1,2-benzisothiazol-3(2H)-one:

O=C1[N;D3][S;D2]c2[c;D2][c;D2][c;D2][c;D2]c12

Strained bicyclobutane:

[C;D4]12[C;D2][C;D3]1[C;D2]2

Strained bicyclopentane:

[C;D4]12[C;D2][C;D3]1[C;D2][C;D2]2

Sulfamoyl fluoride:

[N;D3]S(=O)(=O)F

Sulfonate ester:

[S;D4](=O)(=O)[O;D2]

Sulfonimin/doyl fluoride:

[S;D4](=O)(F)=[N;D2]

Sulfonium methyl amide, ketone:

O=[C;D3][C;D2][S;D3;+][C;D1]

O=C([N;D2;X3])[C;D2][S;D3;+][C;D1]

Sulfonyl acrylate:

[O;D2]C(=O)C(=[C;D1])[C;D2][S;D4](=O)=O

Sulfonyl chloride:

Cl[S;D4](=O)=O

Sulfonyl fluoride:

F[S;D4](=O)=O

Sulphur-triazole exchange (SuTEx):

[S;D4](=O)(=O)N1N=[C;D3]N=[C;D2]1

Thiirane:

[S;D2]1[C;D3;X4][C;D2,D3;X4]1

Thiocarbamate, dithiocarbamate:

[O,S;D2]C(=[S;D1])[N;D2;X3]

Thiol:

[S;D1]

Thiophosphorodichloridate:

[O;D2]P(=[S;D1])(Cl)Cl

References

(1) Bellmann, L.; Klein, R.; Rarey, M. Calculating and Optimizing Physicochemical Property Distributions of Large Combinatorial Fragment Spaces. *J. Chem. Inf. Model.* **2022**, *62*, 2800–2810.

(2) Péczka, N.; Orgován, Z.; Ábrányi Balogh, P.; Keserű, G. M. Electrophilic Warheads in Covalent Drug Discovery: An Overview. *Expert Opin. Drug Discovery* **2022**, *17*, 413–422.
